# *HJURP* promotes proliferation in prostate cancer cells through increasing *CDKN1A* degradation via the *GSK3β/JNK* signaling pathway

**DOI:** 10.1038/s41419-021-03870-x

**Published:** 2021-06-07

**Authors:** Wenjie Lai, Weian Zhu, Chutian Xiao, Xiaojuan Li, Yu Wang, Yuefu Han, Jiayu Zheng, Yingqiu Li, Mingqiang Li, Xingqiao Wen

**Affiliations:** 1 Department of Urology, The Third Affiliated Hospital, Sun Yat-sen University, Guangzhou, China; 2 Department of Health Care, Shenzhen Hospital, Southern Medical University, Shenzhen, China; 3 Department of Urology, Yue Bei People’s Hospital, Shaoguan, China; 4 State Key Laboratory of Biocontrol, Key Laboratory of Gene Engineering of the Ministry of Education, School of Life Sciences, Sun Yat-sen University, Guangzhou, China; 5 Laboratory of Biomaterials and Translational Medicine, The Third Affiliated Hospital, Sun Yat-sen University, Guangzhou, China

**Keywords:** Tumour biomarkers, Prostate cancer, Cell growth, Diseases, Molecular biology

## Abstract

Genes with cross-cancer aberrations are most likely to be functional genes or potential therapeutic targets. Here, we found a total of 137 genes were ectopically expressed in eight cancer types, of which Holliday junction recognition protein (*HJURP*) was significantly upregulated in prostate cancer (PCa). Moreover, patients with higher *HJURP* mRNA and protein levels had poorer outcomes, and the protein levels served as an independent prognosis factor for the overall survival of PCa patients. Functionally, ectopic *HJURP* expression promoted PCa cells proliferation in vitro and in vivo. Mechanistically, *HJURP* increased the ubiquitination of cyclin-dependent kinase inhibitor 1 (*CDKN1A)* via the *GSK3β/JNK* signaling pathway and decreased its stability. This study investigated the role of *HJURP* in PCa proliferation and may provide a novel prognostic and therapeutic target for PCa.

## Introduction

The incidence of prostate cancer (PCa) has continued to rise in recent years and is currently ranked first among all male malignancies in the USA.^1^ Therefore, it is imperative to identify new diagnostic and therapeutic targets for PCa. Many studies have reported that genes with cross-cancer aberrations are most likely to be functional genes and potential therapeutic targets^2,3^. Thus, we analyzed the RNA-seq data of eight cancer types, including PCa, from the FireBrowse database (http://firebrowse.org/) to determine the cross-oncogenes. The results showed that 137 genes are differentially expressed across all tumors assessed, and *HJURP* was most significantly upregulated in data from PCa.

In mammals, *HJURP* is a molecular chaperone of centromere protein-A (*CENP-A*) and mediates the deposition of *CENP-A* in centromeres to promote chromosome separation and mitosis^4^. Furthermore, *HJURP* inhibits senescence through the *p53*-dependent pathway and plays a role in cell viability regulation^5^. Kato et al.^6^ revealed that, in cancer cells, *HJURP* promotes homologous recombination and rDNA stability through interaction with human mutS homolog 5 (*hMSH5*) and *MRE11-RAD50-NBS1* protein complex, which contributes to immortality and genomic stability in osteosarcoma, lung, and testicular cancer. Moreover, Cao et al.^7^ reported that *HJURP*-silencing could activate *PPARγ* and inhibit the *p-SIRT1/t-SIRT1* signaling pathways in bladder cancer cells, which can lead to an increase in ROS and cause apoptosis. In addition, *HJURP* is elevated in ovarian^8^ and breast cancer^9^ and is correlated with poor outcomes.

Similarly, *HJURP* is an unfavorable factor in PCa^10^, but the specific functions and mechanisms underlying its involvement remain unclear. In this study, we demonstrated that *HJURP* was upregulated in PCa and its expression was positively correlated with unfavorable outcomes. Moreover, *HJURP* increased the ubiquitination of *CDKN1A* through the *GSK3β/JNK* signaling pathway, which promoted the proliferation of PCa cells in vitro and in vivo.

## Results

### A total of 137 genes were ectopically expressed in eight cancer types, of which *HJURP* was most significantly upregulated in PCa

**Fig. 1:**
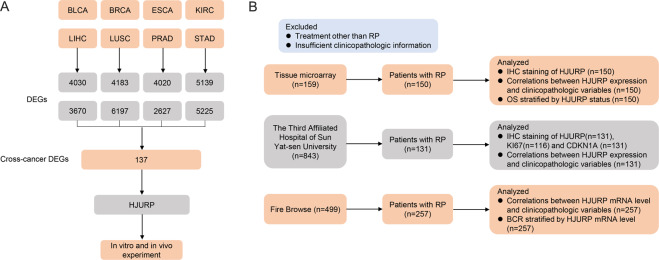
Flowchart depicting study enrollment. **A** Screening of cross-oncogene in eight tumors. **B** The patients with prostate cancer enrolled in this study. BLCA bladder urothelial carcinoma, BRCA breast invasive carcinoma, ESCA esophageal carcinoma, KIRC kidney renal clear cell carcinoma, LIHC liver hepatocellular carcinoma, LUSC lung squamous cell carcinoma, PRAD prostate adenocarcinoma, STAD stomach adenocarcinoma, DEGs differentially expressed genes, RP radical prostatectomy, IHC Immunohistochemistry, OS overall survival, BCR biochemical recurrence.

**Fig. 2:**
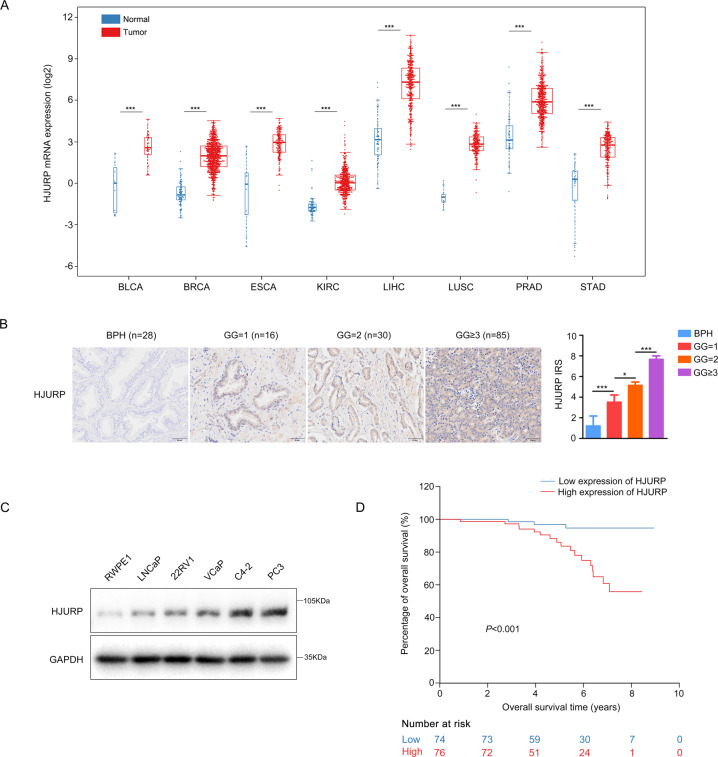
*HJURP* was upregulated in PCa and positively correlated with poor overall survival (OS). **A** *HJURP* mRNA levels were significantly elevated in all eight types of tumors. **B** Representative IHC staining of *HJURP* in PCa and BPH tissues from our hospital (scale bar: 50 µm; magnification: ×200). **C** Western blotting was used to measure *HJURP* expression in different PCa cell lines. **D** Kaplan–Meier plots demonstrating OS time stratified by *HJURP* protein levels. BPH benign prostate hyperplasia, IRS immunoreactivity score, GG Gleason grade.

The analysis of RNA-seq data from FireBrowse showed that a total of 137 genes were differentially expressed across all tumors (Fig. 1A, Supplementary Fig. S1A, and Table S3). In PCa, 137 genes were differentially expressed between tumor and normal tissues (Supplementary Fig. S1B), which *HJURP* was most significantly upregulated in PCa (fold change = 6.2, *P* < 0.05, Supplementary Fig. S1C), and its mRNA levels were substantially elevated among the eight tumors (Fig. 2A). Moreover, IHC staining confirmed that compared with BPH, *HJURP* was notably increased in PCa tissues and positively correlated with Gleason grade (GG) (Fig. 2B and Supplementary Fig. S1D). Western blotting also showed that compared with RWPE1 cells, *HJURP* was significantly upregulated in LNCaP, 22Rv1, VCaP, C4-2, and PC3 cells (Fig. 2C).

These results indicate that *HJURP* is a cross-oncogene, notably upregulated in PCa.

### High *HJURP* mRNA and protein levels are positively correlated with poor outcomes

The IHC staining of PCa specimens from our hospital (*n* = 131) and tissue array (*n* = 150) both showed that *HJURP* protein level was strongly associated with the pathologic GG group (Supplementary Table S4). Moreover, the *HJURP* mRNA levels (*n* = 257) were also positively correlated with the pathologic GG group (Supplementary Table S4). Kaplan–Meier survival analyses from the tissue array indicated that patients with higher *HJURP* protein levels had shorter overall survival (OS) time (*P* < 0.001, Fig. 2D). Furthermore, univariate and multivariate Cox regression analyses showed that high *HJURP* protein expression was an independent prognostic factor for the poor OS of PCa patients (HR = 5.62, 95% CI = 1.53–20.60, *P* = 0.009, Table 1). Moreover, the high *HJURP* mRNA levels were also positively associated with increased biochemical recurrence rate (BCR) after RP (*P* = 0.024) in PCa patients from the FireBrowse database (Supplementary Fig. S1E). However, multivariate Cox regression analyses failed to confirm the *HJURP* mRNA level as an independent prognostic factor for BCR of PCa patients (data not shown).

**Table 1:** Univariate and multivariate Cox regression analyses for overall survival in PCa patients enrolled in tissue microarray.

	Univariable models		Multivariable models		Multivariable models	
Variables	HR (95% CI)	*P* value	HR (95% CI)	*P* value	HR (95% CI)	*P* value
*Age (years)*						
≤65	Ref.		Ref.		Ref.	
>65	11.60 (1.55, 86.75)	0.017	14.11 (1.67, 119.22)	0.015	13.31 (1.63, 108.51)	<0.001
*Gleason grade group at RP*			–		–	
1	Ref.					
2	0.49 (0.04, 5.44)	0.563				
≥3	2.35 (0.31, 17.67)	0.408				
*Pathological T stage*					–	
T2	Ref.		Ref.			
T3a	4.36 (1.47, 12.95)	0.008	0.79 (0.20, 3.10)	0.730		
T3b	9.17 (2.49, 33.86)	0.001	0.78 (0.16, 3.83)	0.758		
*Pathological N stage*						
N0	Ref.		Ref.		Ref.	
N1	14.27 (4.74, 42.95)	<0.001	34.48 (6.67, 178.34)	<0.001	28.32 (7.89, 101.60)	<0.001
*IRS of HJURP*						
≤6	Ref.		Ref.		Ref.	
>6	6.63 (1.94, 22.64)	0.003	5.84 (1.56, 21.85)	0.009	5.62 (1.53, 20.60)	0.009
*Surgical margins*						
Negative	Ref.		Ref.		Ref.	
Positive	6.01 (2.44, 14.84)	<0.001	11.12 (3.60, 34.31)	<0.001	10.38 (3.55, 30.42)	<0.001

*IRS* immunoreactivity score, *RP* radical prostatectomy.

In addition, the T stage of patients from the Third Affiliated Hospital denotes statistically significant differences between the high- and low-expression groups of *HJURP*, but there was no significant difference in patients from the tissue array (Supplementary Table S4). We think it is caused by differences in health care and insurance systems. The Third Affiliated Hospital mainly treats patients from undeveloped areas with limited medical resources and a lack of early screening for PCa, resulting in a higher T stage. However, most of the patients enrolled in the tissue array come from developed regions with better health care, for the timely treatment prevented the PCa from progression.

Overall, the results indicate that high *HJURP* mRNA and protein levels are both significantly correlated with a poor outcome, and *HJURP* protein levels serve as an independent prognosis factor for OS of PCa patients.

### *HJURP* promotes the proliferation of PCa cells in vitro and in vivo

**Fig. 3:**
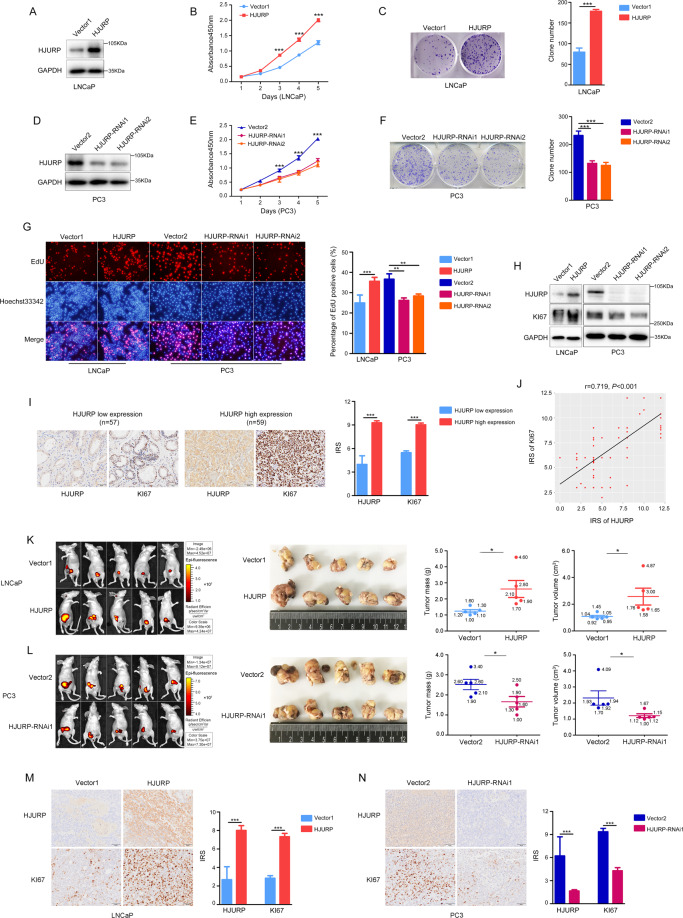
*HJURP* promotes the proliferation of PCa cells in vitro and in vivo. **A**, **D** Western blotting of *HJURP*-overexpression (**A**) and knockdown (**D**) efficiency. **B**, **E** CCK8 assay to measure PCa cells viability after *HJURP*-overexpression (**B**) and knockdown (**E**). **C**, **F** Colony-formation assay to measure the cell viability of PCa cells after *HJURP* overexpression (**C**) and silencing (**F**). **G** EdU assay to measure the effects of *HJURP* on the proliferative capacity of PCa cells. **H** Western blotting quantification of *KI67* expression after *HJURP* overexpression or knockdown in PCa cells. **I** Representative IHC staining of *HJURP* and *KI67* in different Gleason grade PCa tissues from our hospital (*n* = 131, scale bar: 50 µm; magnification: ×200). **J** *HJURP* and *KI67* protein expression are highly correlated in PCa (*n* = 131). **K**, **L** *HJURP* can affect the in vivo growth of PCa cells. **M**, **N** Representative IHC staining of *HJURP* and *KI67* in PCa-cell xenograft tissues (scale bar: 50 µm; magnification: ×200).

To investigate the specific function of *HJURP* in PCa cells, we overexpressed *HJURP* in LNCaP cells (Fig. 3A). A CCK8 assay showed that the viability of LNCaP cells significantly increased (Fig. 3B). Conversely, *HJURP* was silenced in PC3 cells (Fig. 3D), and cell viability was significantly decreased (Fig. 3E). Similar results were also obtained from the colony-formation assay (Fig. 3C, F). In addition, *HJURP* overexpression can significantly increase the proliferative capacity of LNCaP cells, and *HJURP* knockdown inhibited the proliferative capacity of PC3 cells (Fig. 3G). Similarly, *HJURP* knockdown in LNCaP cells and overexpression in PC3 cells have the same results (Supplementary Fig. 2A–G). Western blotting established that *KI67* was positively correlated with *HJURP* (Fig. 3H). Notably, the IHC staining of PCa tissues (*n* = 116) from our hospital also showed a high correlation between *HJURP* and *KI67* (*r* = 0.719, *P* < 0.001, Fig. 3I, J), which provided strong evidence for *HJURP* association with PCa proliferation. However, *HJURP* was weakly affected the apoptosis of LNCaP and PC3 cells (Supplementary Fig. S2H). Consistent with the results described, the in vivo growth of LNCaP cells with stable *HJURP* expression was notably enhanced (Fig. 3K), whereas that of PC3 cells with *HJURP* silencing was significantly impaired (Fig. 3L). IHC staining of the xenograft demonstrated that *KI67* expression was positively correlated with *HJURP* levels (Fig. 3M, N).

In summary, we found that *HJURP* can promote PCa cell proliferation, but not inhibit apoptosis in vitro and in vivo.

### *HJURP* promotes G1/S phase transition through *CDKN1A* inhibition in PCa cells

**Fig. 4:**
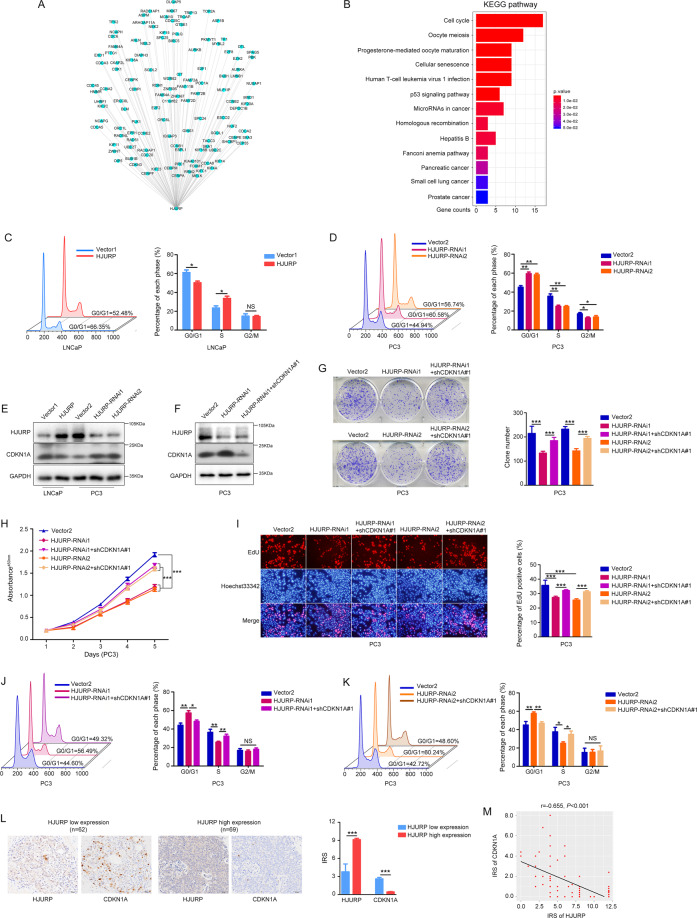
*HJURP* inhibits *CDKN1A* to promote the G1/S phase transition. **A** Correlation analysis showed that 119 genes were highly correlated with *HJURP* (*r* ≥ 0.06, *P* < 0.05). **B** KEGG enrichment analysis of *HJURP* and its highly correlated genes. **C**, **D** Flow cytometry used to measure the proportion of cells in various phases of the cell cycle in *HJURP*-overexpression (**C**) and -knockdown (**D**). **E** Western blotting quantified *CDKN1A* expression after *HJURP-*overexpression or -knockdown in PCa cells. **F** Western blotting results of *CDKN1A* knockdown efficiency. **G**–**K** *CDKN1A* knockdown significantly reversed changes in clonogenic potential (**G**), cell viability (**H**), proliferative capacity (**I**), and cell cycle (**J**, **K**) in PC3 cells caused by *HJURP* inhibition. **L** Representative IHC staining of *HJURP* and *CDKN1A* in different Gleason grade PCa tissues from our hospital (*n* = 131, scale bar: 50 µm; magnification: ×200). **M** *HJURP* and *CDKN1A* protein expression are highly negatively correlated in PCa (*n* = 131).

Genes with similar co-expression patterns may play similar roles in cancer cells^11^. Therefore, co-expression and KEGG enrichment analysis of *HJURP* were performed. The results indicated that a total of 119 genes were highly correlated with *HJURP* (*r* ≥ 0.6, *P* < 0.05, Fig. 4A and Supplementary Table S5) and the cell-cycle pathway had the highest number of enriched genes (Fig. 4B), indicating that *HJURP* may primarily regulate the cell cycle in PCa cells. Consistent with this result, we detected that *HJURP* overexpression promoted G1/S phase transition, whereas *HJURP*-silencing generally induced G0/G1 arrest in PCa cells (Fig. 4C, D and Supplementary Fig. S3A, B).

Chen et al.^12^ reported that *HJURP* could promote hepatocellular carcinoma cell proliferation through the *CDKN1A* protein, a critical factor for G1/S phase transition^13^. Therefore, we hypothesized that *HJURP* also regulates the cell cycle via the *CDKN1A* protein in PCa cells. Western blotting confirmed the hypothesis that *HJURP* overexpression in LNCaP cells decreased *CDKN1A* protein levels, whereas *HJURP* silencing in PC3 cells increased *CDKN1A* protein levels (Fig. 4E). Moreover, CCK8, colony formation, EdU, and flow cytometry assays showed that the impaired proliferative capacity of PC3 cells with *HJURP*-silencing was significantly reversed after *CDKN1A* knockdown (Fig. 4F–K and Supplementary Fig. S3C–3H). More convincingly, *HJURP* expression was negatively correlated with *CDKN1A* levels in PCa tissues (*n* = 131) from our hospital (*r* = −0.655, *P* < 0.001, Fig. 4L, M).

The results show that *HJURP* promotes G1/S phase transition via inhibiting *CDKN1A* protein expression in PCa cells.

### HJURP promotes CDKN1A ubiquitin-dependent proteasome degradation

**Fig. 5:**
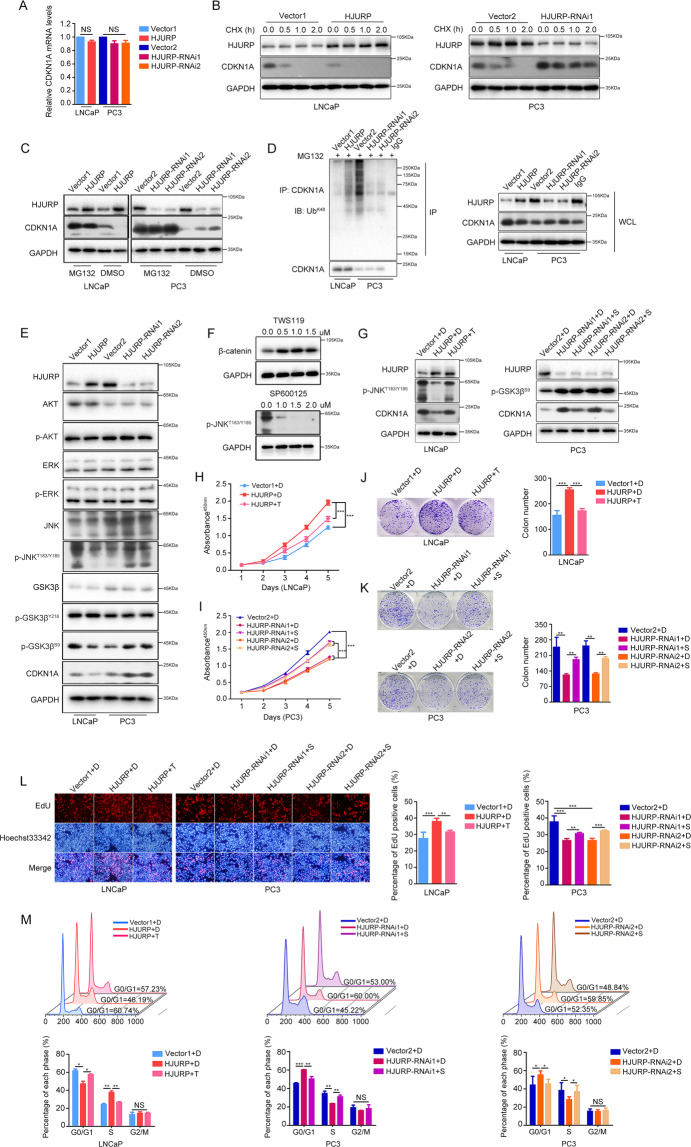
HJURP promotes CDKN1A ubiquitin-dependent proteasomal degradation through the GSK3β/JNK pathway. **A** qPCR quantification of *CDKN1A* mRNA levels in PCa cells after *HJURP*-overexpression or -silencing. **B** CHX (50 µg/mL) was used to treat LNCaP and PC3 cells for 0, 0.5, 1.0, and 2.0 h, and western blotting was applied to quantify *CDKN1A* stability. **C** MG132 (10 µM) was employed to incubate cells for 6 h, and western blotting was utilized to quantify *CDKN1A* levels after *HJURP* -overexpression or -knockdown in PCa cells. **D** CO-IP quantification of the effects of *HJURP* on *CDKN1A* ubiquitination. **E** Western blotting of *AKT*, *p-AKT*, *ERK1/2, p-ERK1/2*, *JNK*, *p-JNK*^*T183/Y185*^, *GSK3β*, *p-GSK3β*^*Y216*^, *p-GSK3β*^*S9*^, and *CDKN1A* levels in PCa cells after *HJURP*-overexpression or -silencing. **F** Western blotting quantified optimal TWS119 and SP600125 concentrations (0.5 µM TWS119 and 1.0 µM SP600125 were selected). **G** TWS119 could reverse *HJURP*-induced reduction in *p-JNK*^*T183/Y185*^ and *CDKN1A*, whereas SP600125 could reverse *HJURP*-induced *CDKN1A* elevation, but did not affect *p-GSK3β*^*S9*^ levels. **H**–**M** TWS119 and SP600125 could reverse *HJURP*-induced changes in cell viability (**H**), clonogenic potential (**J**), proliferative capacity (**L**), and cell cycle (**M**) in PCa cells. CHX cycloheximide, DMSO dimethyl sulfoxide, Ub ubiquitination.

To investigate the specific mechanism by which *HJURP* regulates *CDKN1A*, we first measured the mRNA levels of *CDKN1A* after *HJURP* overexpression and knockdown. The results showed that *HJURP* did not affect *CDKN1A* mRNA transcription (Fig. 5A), indicating that *HJURP* may affect *CDKN1A* expression through post-transcriptional regulation. To validate this hypothesis, we treated PCa cells with a translation inhibitor, cycloheximide (CHX). The results demonstrated that *HJURP* overexpression in LNCaP cells accelerated the degradation of *CDKN1A* protein, whereas *HJURP*-silencing in PC3 cells decreased its degradation rate (Fig. 5B). In addition, the proteasome inhibitor, MG132, significantly reversed *CDKN1A* protein regulation by *HJURP* (Fig. 5C). Together, these results suggest that *HJURP* promotes *CDKN1A* protein degradation through the proteasomal pathway. However, the *CDKN1A* protein can be degraded by ubiquitin-dependent or -independent proteasomal pathways;^14^ therefore, we further tested whether *HJURP* affected *CDKN1A* ubiquitination levels. Protein co-immunoprecipitation showed that the ectopic expression of *HJURP* increased *CDKN1A* ubiquitination levels, whereas *HJURP*-silencing significantly decreased *CDKN1A* ubiquitination levels (Fig. 5D).

In summary, *HJURP* can activate ubiquitin-dependent proteasomal degradation of the *CDKN1A* protein.

### *HJURP* regulates *CDKN1A* ubiquitination through the *GSK3β/JNK* signaling pathway

The ubiquitination of *CDKN1A* protein is affected by its phosphorylation status. For example, extracellular signal-regulated kinase 1/2 (*ERK1/2*)^15^, glycogen synthase kinase (*GSK*) *3β*^16^, c-Jun N-terminal kinase (*JNK*)^17^, and *AKT*^18^ can regulate *CDKN1A* ubiquitination through different phosphorylation sites. Therefore, we hypothesized that *HJURP* might regulate *CDKN1A* ubiquitination through these kinases. Western blotting demonstrated that *HJURP* overexpression decreased *GSK3β S9*, and *JNK T183/Y185* phosphorylation levels, whereas *HJURP*-silencing increased phosphorylation levels at these sites (Fig. 5E). Moreover, many studies have reported that *GSK3β* can act upstream of *JNK* to exert positive or negative regulation^19–21^. Thus, to determine the specific relationship between *GSK3β* and *JNK*, we used TWS119 to inhibit *GSK3β* activity and SP600125 to inhibit *JNK* activity (Fig. 5F). The results showed that TWS119 significantly reversed *JNK T183/Y185* phosphorylation and *CDKN1A* protein levels in *HJURP*-overexpression LNCaP cells and enhanced that in *HJURP*-knockdown PC3 cells, however, SP600125 failed to affect the phosphorylation level of *GSK3β S9* in both cells (Fig. 5G and Supplementary Fig. S4A). These results suggest that *HJURP* regulates *CDKN1A* ubiquitination through the *GSK3β/JNK* signaling pathway. Based on the results of CCK8, colony formation, Edu, and flow cytometry assay, the growth affected by *HJURP* were significantly reversed by treatment with TWS119 and SP600125 in *HJURP* ectopic expression-LNCaP cells and *HJURP*-knockdown PC3 cells, respectively (Fig. 5H–M). Moreover, the proliferation ability of *HJURP*-overexpression LNCaP cells was enhanced by SP600125, and that of *HJURP*-knockdown PC3 cells were impaired by TWS119 (Supplementary Fig. S4B–4G).

**Fig. 6:**
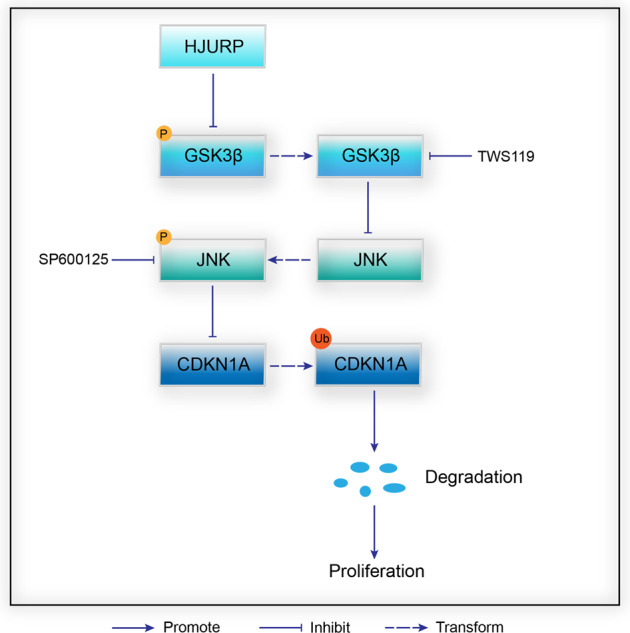
Mechanisms of *HJURP* in PCa cells. *HJURP* inhibits the phosphorylation of GSK3β S9 and JNK T183/Y185, which activates the ubiquitin-dependent proteasomal degradation pathway of CDKN1A and ultimately promotes the proliferation of PCa cells. P phosphorylation, Ub ubiquitination.

Taken together, *HJURP* promotes ubiquitin-dependent proteasome degradation of *CDKN1A* via the *GSK3β/JNK* signaling pathway (Fig. 6).

## Discussion

In this study, we discovered 137 cross-oncogenes from eight types of tumors, of which *HJURP* was the most significantly upregulated gene for PCa. Moreover, *HJURP* protein levels were an independent prognostic factor for short OS time. Furthermore, *HJURP* can promote G1/S phase transition through increasing degradation of *CDKN1A* protein via the *GSK3β/JNK* signaling pathway, which leads to proliferation of PCa cells in vitro and in vivo.

It is well-known that the stability of *CDKN1A*, a crucial factor for transition from G1 to S phase^22^, depends on its phosphorylation status^23^. *GSK3β* is a *CDKN1A* kinase that phosphorylates different sites to promote ubiquitin-dependent or -independent proteasomal degradation of *CDKN1A*^14,24^. Conversely, *JNK* can decrease *CDKN1A* ubiquitination levels to increase its stability^13,25^. Moreover, *GSK3β* can also regulate *JNK* as an upstream factor: Liu et al.^19^ found that *GSK3β* is a negative regulator of growth factor-induced activation of *JNK* in mouse embryonic fibroblasts. Abell et al.^20^ reported that *GSK3β* binds to the C-terminal kinase domain of *MEKK4* in COS-7 cells, which blocks *MEKK4* dimerization and ultimately inhibits *JNK* activity. Furthermore, Yuri et al.^21^ reported that in glioblastoma cells, *JNK* phosphorylation levels decreased after *GSK3β* was inhibited. These studies demonstrated that *GSK3β* could positively or negatively regulate *JNK* as an upstream factor. In this study, *HJURP* could activate *GSK3β* while inhibiting *JNK* activity in PCa cells. Moreover, treating *HJURP*-overexpression PCa cells with TWS119 could significantly reverse *p-JNK*^*T183/Y185*^ levels, however, the expression of *p-GSK3β*^*S9*^ did not change after incubation with SP600125 in *HJURP*-knockdown PCa cells. Thus, we conclude that *HJURP* inhibits *CDKN1A* expression via the *GSK3β/JNK* signaling pathway, which promotes the proliferation of PCa cells in vitro and in vivo ultimately.

Interestingly, the subcellular location *of HJURP* was initially reported to be nucleus^4^, however, we found that it was mainly stained in the cytoplasm of PCa cells according to the IHC. This is consistent with prior studies in PCa^10^. In addition, in hepatocellular carcinoma^12,26^, *HJURP* is also stained in the cytoplasm. However, in pancreatic cancer^27^ and colorectal cancer^28^, its localization is mainly in the nucleus. In glioma^29^, *HJURP* is stained in both cytoplasm and nucleus. These results suggested that the subcellular localization of *HJURP* varies in different kinds of cancer, and different localization of *HJURP* may lead to different functions, but the specific mechanism needs further investigation.

In conclusion, our findings revealed that *HJURP*, a cross-oncogene, negatively correlated with outcomes of PCa patients, promoted proliferation in PCa cells via inhibition of *CDKN1A* expression through the *GSK3β/JNK* signaling pathway. These findings may provide a novel prognostic and therapeutic target for PCa.

## Materials and methods

### Analysis of RNA-seq and clinical data from public database

The RNA-seq data of eight types of cancer with the most comprehensive information were downloaded from the FireBrowse database (http://firebrowse.org/) (Fig. 1A and Supplementary Table S1). The R 3.5.1 and Perl 5.24.1 were employed to analyze the data.

Clinicopathological information from 499 PCa patients was obtained from the FireBrowse database; 257 patients who underwent radical prostatectomy (RP) provided sufficient information to be included in the present analysis (Fig. 1B and Supplementary Tables S1 and S2), the detailed inclusion and exclusion criteria are exhibited in Supplementary Methods. Patients were divided into high-expression and low-expression groups according to the median RNA-Seq by Expectation-Maximization (RSEM) of *HJURP* mRNA.

### Immunohistochemical (IHC) staining

The tissue array was purchased from Wuhan Servicebio Biotech Co. (Shanghai, China). Moreover, PCa tissue specimens of 131 patients and benign prostate hyperplasia (BPH) tissue specimens of 28 patients were obtained from the Third Affiliated Hospital of Sun Yat-sen University, Guangzhou, China (Fig. 1B). The inclusion and exclusion criteria of tissue array and specimens were presented in Supplementary Methods. Patient characteristics from our study cohort are displayed in Supplementary Table S2. This study was approved by the Ethics Review Committee of the Third Affiliated Hospital of Sun Yat-sen University and registered in the Chinese Clinical Trial Registry (ChiCTR2000033835). Written informed consent was received from patients according to the guidelines of the Declaration of Helsinki. IHC staining was based on the methods described previously^30^. The primary antibodies included *HJURP* (Abcam, ab175577, USA), *KI67* (Abcam, ab16667, USA), and *CDKN1A* (CST, 2947, USA). The tissue samples were evaluated according to immunoreactive score (IRS). IRS > 6 was defined as a high expression of *HJURP* while IRS ≤ 6 was defined as a low expression of *HJURP*.

IRS = staining intensity × percentage of stained cells. Staining intensity: negative, 0; weak, 1; moderate, 2; strong, 3; percentage of stained cells: 0%, 0; <10%, 1; 11–50%, 2; 51–80%, 3; >80%, 4.

### Cell culture

LNCaP and PC3 cells were purchased from the American Type Culture Collection (Manassas, USA). LNCaP and PC3 cells were cultured in RPMI-1640 (HyClone, USA) with 10% fetal bovine serum (FBS; Bovogen, Australia). All cells were incubated in a humidified incubator at 37 °C with 5% CO_2_. All cells used in this study were identified by short tandem repeat authentication and excluded the mycoplasma contamination.

### Construction and transfection of plasmids and lentivirus

Lentivirus: *HJURP*-overexpressing and knockdown lentiviruses and control viruses were purchased from OBiO Technology Corporation (Shanghai, China). *HJURP*-overexpressing and control lentiviruses were transfected into LNCaP cells, *HJURP* knockdown and control lentiviruses were transfected into PC3 cells according to the manufacturer’s instructions. Puromycin at 3 µg/mL (LNCaP) or 5 µg/mL (PC3) was employed to screen stably transfected cells; *HJURP*-RNAi1, 5’-GTATGGAAGTTCGATATCA-3’; *HJURP*-RNAi2, 5’-GTGACACCCTCGAAGTATT-3’.

Plasmids: *CDKN1A*-shRNA was constructed using pSUPER.retro.neo vector, and the empty vector was regarded as the control. Lipofectamine 3000 (Invitrogen, USA) was used for transfection according to the manufacturer’s instructions; sh*CDKN1A#1*: 5’- GATCCCCGATGGAACTTCGACTTTGTTTCAAGAGACTACCTTGAAGCTGAAACATTTTTA-3’; sh*CDKN1A#2*: 5’-GATCCCCGAGACTCTCAGGGTCGAAATTCAAGAGACTCTGAGAGTCCCAGCTTTTTTTTA-3’.

### Western blotting

Western blotting was performed as previously described^30^. Primary antibodies included those for *HJURP* (Abcam, ab100800, USA), *KI67* (Abcam, ab16667, USA), *CDKN1A* (CST, 2947, USA), *AKT* (CST, 4691, USA), *p-AKT*^*T308*^ (CST, 13038, USA), *ERK1/2* (CST, 4695, USA), *p-ERK1/2* (CST, 4370, USA), *JNK* (CST, 9252, USA), *p-JNK*^*T183/Y185*^ (CST, 4668, USA), *GSK3β* (Abcam, ab93926, USA), *p-GSK3β*^*Y216*^ (Abcam, ab75745, USA), *p-GSK3β*^*S9*^ (CST, 9322, USA), *β-catenin* (CST, 8480, USA), and *GAPDH* (ABclonal, A19056, China). Other reagents included cycloheximide, MG132, TWS119, and SP600125 (Sigma).

### Cell viability assay

The Cell Counting Kit-8 Assay (Dojindo, Japan) was used to measure cell viability.

To investigate whether *HJURP* affected the viability of PCa cells, LNCaP (3 × 10^3^ cells/well) and PC3 cells (2 × 10^3^ cells/well) were seeded into 96-well plates and cultured in a humidified incubator at 37 °C with 5% CO_2_ for 1–5 days. After the indicated number of days, the supernatants were replaced by fresh culture medium containing 10% CCK8 and then incubated for an hour at 37 °C. A microplate reader (Bio Rad, USA) was used to measure absorbance at 450 nm.

### Colony-formation assay

Stably infected LNCaP and PC3 cells were seeded into six-well plates at a density of 1 × 10^3^ cells/well. After incubation for 14 days, the plates were washed with phosphate-buffered saline (PBS) three times, and 4% paraformaldehyde was applied for 15 min to fix the cells. Subsequently, 0.5% crystal violet solution (KeyGEN, China) was used to stain the cells. After 10 min, the plates were washed with pure water for further counting and analysis.

### EdU assay

The cells were seeded in 12-well plates and incubated with EdU (Ribobio, C10310, China) for 4 h at 37 °C with 5% CO_2_. Subsequently, the supernatants were removed and 500 µl of 1 × Apollo^®^ reaction cocktail was employed to treat cells for 30 min. Following this, the DNA contents of the cells were stained with 500 µl of Hoechst33342 (5 µg/ml) for 30 min and detected under a fluorescence microscope (Olympus Optical, Japan).

### Mouse xenograft assay

Four- to six-week-old BALB/c nude male mice were purchased from the Laboratory Animal Center of Sun Yat-sen University (Guangzhou, China), were used in this study. Mice were housed in a specific pathogen-free environment with sterile food and water. All animal experiments underwent the necessary ethical review (SYSU-IACUC-2020-000202) and were performed with strict adherence to the regulations of the Sun Yat-Sen University Animal Care and Use Committee.

The sample size of mice was decided from previous reports^30^ and practical considerations, blinding and randomization method were employed to the group.

After anesthetizing the mice with sodium pentobarbital (40 mg/kg) (Sigma), LNCaP cells stably expressing *HJURP* (5 × 10^6^ cells/ mice) or PC3 cells with stable *HJURP*-silencing (1 × 10^6^ cells/ mice) and their control cells, resuspended in 100 µl Hanks’ balanced salt solution (HBSS, HyClone), were injected into the prostate with a 29-gauge needle (These mice were divided into four groups: LNCaP-Vector1, LNCaP-*HJURP*, PC3-Vector2, and PC3-*HJURP*-RNAi1, with five mice/group), of which the bilateral testes of mice in the PC3 group were simultaneously resected. After 4 weeks, mice were anesthetized and imaged using an IVIS system (PerkinElmer, USA). Subsequently, mice were euthanized and tumors were extracted for further histomorphological analysis. The carcasses were disposed of by the Laboratory Animal Center.

### Gene co-expression and KEGG enrichment analysis

To obtain genes highly correlated with *HJURP* (*r* ≥ 0.6, *P* < 0.05), RNA-seq data from PCa cases emanating from FireBrowse were analyzed using Hmisc package in R 3.5.1. Then, the clusterProfiler package in R 3.5.1 was applied for KEGG enrichment analysis of the genes.

### Flow cytometry

Cell-cycle analysis: Pre-cooled 75% ethanol was used to fix PCa cells (1 × 10^6^ cells) at 4 °C overnight. Subsequently, the cells were washed with PBS three times and resuspended with 500 µl propidium iodide (PI) (Becton, Dickinson and Company, USA) at room temperature. After 15 min, the cells were loaded into the flow cytometer (Calibur, BD Bioscience, USA) for analysis. FlowJo software (Tree Star, San Carlos, USA) was used to evaluate the results.

Cell apoptosis analysis: The PCa cells (1 × 10^6^ cells) were washed with pre-cooled PBS twice and resuspended in 1 ml of 1× binding buffer. Then, 5 µl FITC Annexin V and 5 µl PI (Becton, Dickinson and Company) were added to 100 µl of cell suspension (1 × 10^5^ cells), and the cells were incubated for 15 min at room temperature. Finally, 200 µl of 1× binding buffer was added to each tube, and cell apoptosis analysis was detected via a flow cytometer (Calibur, BD Bioscience). FlowJo software (Tree Star) was used to analyze the results.

### RT-qPCR

Trizol (Invitrogen) was employed to extract total RNA from PCa cells, and superscript III reverse transcriptase (Invitrogen) was used for the reverse transcription of 1 µg of the total RNA. SYBR Green qPCR reagent (GenStar, China) was used for fluorescence real-time PCR, and the mRNA levels of GAPDH were utilized as the internal reference genes.

qPCR primers: *CDKN1A*, upstream primer: 5’-CGATGGAACTTCGACTTTGTCA-3’, downstream primer: 5’-GCACAAGGGTACAAGACAGTG-3’; *GAPDH*, upstream primer: 5’-GACTCATGACCACAGTCCATGC-3’, downstream primer: 5’-AGAGGCAGGGATGATGTTCTG-3’.

### Co-immunoprecipitation and ubiquitination assays

The PCa cells were seeded into six-well plates, and the supernatants were replaced with fresh culture medium containing 10 µM/L MG132 when the cell density was 70–80%. After 6 h, the cells were washed with PBS three times and lysed in 120 µl lysis buffers (containing 0.1% protease inhibitor, 1% phosphatase inhibitor, and 1% phenylmethanesulfonyl fluoride). The homogenates were incubated on ice for 30 min and the samples were centrifuged at 16,000 × *g* for 10 min at 4 °C. Then, 25 µl of whole-cell lysates were used for western blotting, and the remaining 95 µl of lysates were incubated at 95 °C for 10 min with 5 µl of 20% SDS. Subsequently, the cell lysates were used for immunoprecipitation with *CDKN1A* (CST) or IgG primary antibodies (Abcam, ab6715, USA), and the samples were incubated at 4 °C in a shaking incubator. After 5 h, protein-A/G mix beads (Thermo Scientific) were added and the samples were incubated at 4 °C overnight. Following this, the immunoprecipitates were collected and washed with lysis buffer three times and prepared for western blotting. Ubiquitination antibodies (Millipore, 05-1307, USA) were used for the detection of *CDKN1A* ubiquitination levels.

### Statistical analysis

All experiments were performed in triplicate. The R 3.5.1, Perl 5.24.1, and the Statistical Package for the Social Sciences (SPSS) v.22.0 software packages (SPSS Inc., Chicago, USA) were used to perform statistical analyses, and the data were expressed as mean ± standard deviation (SD). The relationship between *HJURP* and clinicopathological parameters was analyzed using *χ*^2^ test or Fisher’s exact test, whenever appropriate. The correlations between *HJURP* and OS were assessed using the log-rank test for Kaplan–Meier methods, and the median IRS of *HJURP* was used as the cutoff point to separate the samples into high- and low-expression groups. The median RNA-Seq by RSEM of *HJURP* mRNA was the cutoff point dividing the patients from FireBrowse into high- and low-level groups when assessed the correlation between *HJURP* and BCR. OS data were analyzed using univariate analysis initially, and covariates with a *P* value ≤0.05 were enrolled in multivariate Cox regression analysis subsequently. The results of the CCK8 assay, colony-formation assay, EdU assay, cell-cycle and apoptosis flow cytometry, and animal experiments were evaluated with independent-sample *t* test. A *P* < 0.05 was considered to be statistically significant. * represents *P* < 0.05, ** represents *P* < 0.01, and *** represents *P* < 0.001.

## Supplementary information

Supplementary Tables S1

Supplementary Tables S2

Supplementary Tables S3

Supplementary Tables S4

Supplementary Tables S5

Supplementary Fig. S1

Supplementary Fig. S2

Supplementary Fig. S3

Supplementary Fig. S4

Supplemental Figure legends

Supplementary Methods

## Data Availability

All data and materials generated and/or analyzed during the current study are available from the corresponding author upon reasonable request.

## Code availability

The related computerized programs for the analysis using R or Perl are exhibited in Supplementary Methods.
