# Supplementary material for: HJURP promotes proliferation in prostate cancer cells through increasing CDKN1A degradation via the GSK3β/JNK signaling pathway
Source: Cell Death Dis. 2021 Jun 7;12(6):583. doi: 10.1038/s41419-021-03870-x (PMC8184824; doi:10.1038/s41419-021-03870-x)
Supplement: Supplementary file 1 — Supplementary Tables S1 [file 41419_2021_3870_MOESM1_ESM.docx]

**Table S1. The information of RNA-seq and clinical features from FIREBROWSE database**

| **Tumor** | **File name of RNA seq data** | **Data** | **File name of clinical data** | **Clinical features** | **Sample**  **(Tumor/Normal)** |
| --- | --- | --- | --- | --- | --- |
| STAD | STAD.rnaseq__illuminahiseq_rnaseq__bcgsc_ca__Level_3__gene_expression__data.data | RPKM | NA | NA | 237/33 |
| BLCA | BLCA.rnaseq__illuminahiseq_rnaseq__unc_edu__  Level_3__gene_expression__data.data | RPKM | NA | NA | 55/12 |
| BRCA | BRCA.rnaseq__illuminahiseq_rnaseq__unc_edu__Level_3__gene_expression__data.data.txt | RPKM | NA | NA | 774/87 |
| ESCA | ESCA.rnaseq__illuminahiseq_rnaseq__bcgsc_ca__Level_3__gene_expression__data.data.txt | RPKM | NA | NA | 183/13 |
| KIRC | KIRC.rnaseq__illuminahiseq_rnaseq__unc_edu__Level_3__gene_expression__data.data | RPKM | NA | NA | 468/68 |
| LIHC | LIHC.rnaseqv2__illuminahiseq_rnaseqv2__unc_edu__Level_3__RSEM_genes_normalized__data.data | RSEM | NA | NA | 371/53 |
| LUSC | LUSC.rnaseq__illuminahiseq_rnaseq__unc_edu__Level_3__gene_expression__data.data | RPKM | NA | NA | 222/17 |
| PRAD | PRAD.rnaseqv2__illuminahiseq_rnaseqv2__unc_edu__Level_3__RSEM_genes_normalized__data.data | RSEM | PRAD.clin.merged | “patient.bcr_patient_barcode”  “patient.age_at_initial_pathologic_diagnosis”  “patient.tumor_samples.tumor_sample.method_of_sample_procurement”  “patient.tumor_samples.tumor_sample.other_method_of_sample_procurement”  “patient.stage_event.tnm_categories.pathologic_categories.pathologic_n”  “patient.stage_event.tnm_categories.pathologic_categories.pathologic_t”  “patient.tumor_samples.tumor_sample.disease_details.gleason_score_combined”  “patient.tumor_samples.tumor_sample.disease_details.gleason_score_primary”  “patient.tumor_samples.tumor_sample.disease_details.gleason_score_secondary”  “patient.tumor_samples.tumor_sample.disease_details.psa_result_preop”  “patient.days_to_last_followup”  “patient.follow_ups.follow_up.days_to_last_followup”  “patient.follow_ups.follow_up-2.days_to_last_followup”  “patient.days_to_first_biochemical_recurrence”  “patient.follow_ups.follow_up.days_to_first_biochemical_recurrence”  “patient.follow_ups.follow_up2.days_to_first_biochemical_recurrence” | 498/52  499 tumors had clinical data but only 257 tumors with detailed information |

STAD, Stomach adenocarcinoma; BLCA, Bladder Urothelial Carcinoma; BRCA, Breast invasive carcinoma; ESCA, Esophageal carcinoma; KIRC, Kidney renal clear cell carcinoma; LIHC, Liver hepatocellular carcinoma; LUSC, Lung squamous cell carcinoma; PRAD, Prostate adenocarcinoma；RPKM, Reads Per Kilobase of exon model per Million mapped reads; RSEM, RNA-Seq by Expectation Maximization
