# Supplementary material for: HJURP promotes proliferation in prostate cancer cells through increasing CDKN1A degradation via the GSK3β/JNK signaling pathway
Source: Cell Death Dis. 2021 Jun 7;12(6):583. doi: 10.1038/s41419-021-03870-x (PMC8184824; doi:10.1038/s41419-021-03870-x)
Supplement: Supplementary file 2 — Supplementary Tables S2 [file 41419_2021_3870_MOESM2_ESM.docx]

**Table S2. Clinicopathological features of the study cohort**

| **Characteristics**  All cases | **The Third Affiliated Hospital** |  | **Tissue microarray** |  | **Fire Browse** |
| --- | --- | --- | --- | --- | --- |
|  | 131 (100.0%) |  | 150 (100.0%) |  | 257 (100.0%) |
| Age in years, median (IQR) | 70 (64, 76) |  | 68 (64, 73) |  | 62 (56, 66) |
| Preoperative PSA (ng/ml), median (IQR) | 19.9 (8.8, 41.7) |  | NA |  | 8.0 (5.3, 12.8) |
| Gleason grade group at RP |  |  |  |  |  |
| 1 | 16 (12.2%) |  | 10 (6.7%) |  | 19 (7.4%) |
| 2 | 30 (22.9%) |  | 47 (31.3%) |  | 88 (34.2%) |
| ≥3 | 85 (64.9%) |  | 93 (62.0%) |  | 150 (58.4%) |
| Pathological T stage |  |  |  |  |  |
| T2 | 31 (23.7%) |  | 123 (82.0%) |  | 92 (35.8%) |
| T3a | 11 (8.4%) |  | 22 (14.7%) |  | 85 (33.1%) |
| T3b | 42 (32.1%) |  | 5 (3.3%) |  | 72 (28.0%) |
| T4 | 1 (0.8%) |  | NA |  | 8 (3.1%) |
| Pathological N stage |  |  |  |  |  |
| Nx | 28 (21.4%) |  | NA |  | NA |
| N0 | 71 (54.2%) |  | 143 (95.3%) |  | 208 (80.9%) |
| N1 | 32 (24.4%) |  | 7 (4.7%) |  | 49 (19.1%) |
| Surgical margins |  |  |  |  |  |
| Negative | 96 (73.3%) |  | 132 (88.0%) |  | NA |
| Positive | 35 (26.7%) |  | 18 (12.0%) |  | NA |
| Prostate volume (cm^3^), median (IQR) | 41.6 (31.7, 58.5) |  | NA |  | NA |
| PSA density, median (IQR) | 0.4 (0.2, 0.9) |  | NA |  | NA |

IQR, interquartile range; PSA, prostate-specific antigen; RP, radical prostatectomy; IRS, immunoreactivity score.
