# Supplementary material for: HJURP promotes proliferation in prostate cancer cells through increasing CDKN1A degradation via the GSK3β/JNK signaling pathway
Source: Cell Death Dis. 2021 Jun 7;12(6):583. doi: 10.1038/s41419-021-03870-x (PMC8184824; doi:10.1038/s41419-021-03870-x)
Supplement: Supplementary file 3 — Supplementary Tables S3 [file 41419_2021_3870_MOESM3_ESM.docx]

**Table S3. The common differentially expressed genes in eight types of tumor**

| **Gene** | **Gene** | **Gene** | **Gene** | **Gene** | **Gene** | **Gene** |
| --- | --- | --- | --- | --- | --- | --- |
| SKA3 | CA4 | ASF1B | C5orf4 | ACACB | FAM111B | C2orf40 |
| NCAPH | B3GNT4 | TOP2A | DLGAP5 | CDH19 | PVT1 | CPNE7 |
| IQGAP3 | BIRC5 | RAD54L | **HJURP** | PGM5 | KIAA0101 | TRIP13 |
| HSPB6 | PLK1 | CDC25C | E2F2 | ERCC6L | MAMDC2 | CFD |
| DPT | ANO5 | CDC45 | SCN7A | KIF2C | AGMAT | PTTG1 |
| CHRDL1 | UBE2C | CNTFR | AURKB | KIF20A | PTH1R | NEK2 |
| TPX2 | FOXM1 | PRR7 | PEG3 | NEGR1 | NTRK3 | TROAP |
| WISP2 | GSG2 | AQP4 | PCDH9 | MCM10 | OIP5 | CENPM |
| ASXL3 | EZH2 | PDZRN4 | CCL21 | MASP1 | CCNA2 | CDCA5 |
| MLF1IP | CCNB2 | SPC25 | MKI67 | TLCD1 | TTK | FAM64A |
| RGN | PLEKHN1 | WDR62 | ACTG2 | GSTM5 | TMEM132A | SPC24 |
| KCNK3 | WNT2 | KIFC1 | TMEM132C | FAM72D | GTSE1 | CENPF |
| BAI3 | PRC1 | FAM72A | SGOL1 | CHTF18 | C7 | ANGPTL1 |
| ASPM | GPRIN1 | PKMYT1 | CDCA2 | F12 | CDKN3 | RDM1 |
| BIK | NEIL3 | MYBL2 | ADH1B | PI16 | BMP5 | MMP9 |
| OGN | CEP55 | KIF15 | FERMT1 | CENPA | BUB1 | MMP11 |
| SCARA5 | FAM72B | KIF14 | HMMR | MELK | POLQ | CDCA8 |
| PTGDS | TRIM29 | FHL1 | KIF4A | CKMT2 | PBK |  |
| TCEAL2 | KIF18B | BUB1B | DES | CDC20 | NUF2 |  |
| CDCA3 | RRM2 | PGR | EXO1 | UHRF1 | NCAPG |  |
