# Supplementary material for: HJURP promotes proliferation in prostate cancer cells through increasing CDKN1A degradation via the GSK3β/JNK signaling pathway
Source: Cell Death Dis. 2021 Jun 7;12(6):583. doi: 10.1038/s41419-021-03870-x (PMC8184824; doi:10.1038/s41419-021-03870-x)
Supplement: Supplementary file 4 — Supplementary Tables S4 [file 41419_2021_3870_MOESM4_ESM.docx]

**Table S4. Association of HJURP expression with clinicopathological features in PCa patients**

|  | **The Third Affiliated Hospital (n=131)** | | |  | **Tissue microarray (n=150)** | | |  | **Fire Browse (n=257)** | | |
| --- | --- | --- | --- | --- | --- | --- | --- | --- | --- | --- | --- |
|  | **HJURP expression** | | |  | **HJURP expression** | | |  | **HJURP mRNA expression** | | |
| **Characteristics** | **Low no. (%)**  **(n=62)** | **High no. (%)**  **(n=69)** | ***P* value** |  | **Low no. (%)**  **(n=74)** | **High no. (%)**  **(n=76)** | ***P* value** |  | **Low no. (%)**  **(n=128)** | **High no. (%)**  **(n=129)** | ***P* value** |
| Age in years, median (IQR) | 69 (63, 75) | 71 (64, 76) | 0.576 |  | 67 (64, 70) | 70 (65, 76) | 0.009 |  | 61 (56, 66) | 63 (57, 67) | 0.216 |
| Preoperative PSA (ng/ml) |  |  | 0.060 |  |  |  | NA |  |  |  | 0.052 |
| <10 | 21 (33.9%) | 15 (21.7%) |  |  | NA | NA |  |  | 88 (68.8%) | 78 (60.5%) |  |
| ≥10<20 | 17 (27.4%) | 13 (18.8%) |  |  | NA | NA |  |  | 28 (21.9%) | 25 (19.4%) |  |
| ≥20 | 24 (38.7%) | 41 (59.4%) |  |  | NA | NA |  |  | 12 (9.3%) | 26 (20.1%) |  |
| Gleason grade group at RP |  |  | <0.001 |  |  |  | 0.002 |  |  |  | <0.001 |
| 1 | 15 (24.2%) | 1 (1.4%) |  |  | 9 (12.2%) | 1 (1.3%) |  |  | 14 (10.9%) | 5 (3.9%) |  |
| 2 | 23 (37.1%) | 7 (10.1%) |  |  | 28 (37.8%) | 19 (25.0%) |  |  | 54 (42.2%) | 34 (26.3%) |  |
| ≥3 | 24 (38.7%) | 61 (88.4%) |  |  | 37 (50.0%) | 56 (73.7%) |  |  | 60 (46.9%) | 90 (69.8%) |  |
| Pathological T stage |  |  | 0.014 |  |  |  | 0.885 |  |  |  | <0.001 |
| T2 | 42 (67.7%) | 29 (42.0%) |  |  | 62 (83.8%) | 61 (80.3%) |  |  | 58 (45.3%) | 34 (26.4%) |  |
| T3a | 6 (9.7%) | 9 (13.0%) |  |  | 10 (13.5%) | 12 (15.8%) |  |  | 44 (34.4%) | 41 (31.8%) |  |
| T3b | 14 (22.6%) | 30 (43.5%) |  |  | 2 (2.7%) | 3 (3.9%) |  |  | 24 (18.8%) | 48 (37.2%) |  |
| T4 | 0 (0.0%) | 1 (1.4%) |  |  | NA | NA |  |  | 2 (1.5%) | 6 (4.6%) |  |
| Pathological N stage* |  |  | 0.104 |  |  |  | 1.000 |  |  |  | 0.008 |
| N0 | 32 (78.0%) | 39 (62.9%) |  |  | 71 (95.9%) | 72 (94.7%) |  |  | 112 (87.5%) | 96 (74.4%) |  |
| N1 | 9 (22.0%) | 23 (37.1%) |  |  | 3 (4.1%) | 4 (5.3%) |  |  | 16 (12.5%) | 33 (25.6%) |  |
| Surgical margins |  |  | 0.071 |  |  |  | 0.345 |  |  |  |  |
| Negative | 50 (80.6%) | 46 (66.7%) |  |  | 67 (91.4%) | 65 (84.6%) |  |  | NA | NA |  |
| Positive | 12 (19.4%) | 23 (33.3%) |  |  | 7 (8.6%) | 11 (15.4%) |  |  | NA | NA |  |
| PSA density |  |  | 0.093 |  |  |  | NA |  |  |  |  |
| <0.15 | 14 (22.6%) | 8 (11.6%) |  |  | NA | NA |  |  | NA | NA |  |
| ≥0.15 | 48 (77.4%) | 61 (88.4%) |  |  | NA | NA |  |  | NA | NA |  |

PSA, prostate-specific antigen; RP, radical prostatectomy

* The Nx stage with 28 patients from The Third Affiliated Hospital had been deleted in this analysis
