# Supplementary material for: HJURP promotes proliferation in prostate cancer cells through increasing CDKN1A degradation via the GSK3β/JNK signaling pathway
Source: Cell Death Dis. 2021 Jun 7;12(6):583. doi: 10.1038/s41419-021-03870-x (PMC8184824; doi:10.1038/s41419-021-03870-x)
Supplement: Supplementary file 5 — Supplementary Tables S5 [file 41419_2021_3870_MOESM5_ESM.docx]

**Table S5. Genes show high correlation with HJURP in prostate cancer**

| **Gene** | **r** | **Gene** | **r** | **Gene** | **r** | **Gene** | **r** | **Gene** | **r** | **Gene** | **r** |
| --- | --- | --- | --- | --- | --- | --- | --- | --- | --- | --- | --- |
| CENPK | 0.60 | ESCO2 | 0.67 | CDC6 | 0.73 | FAM72B | 0.75 | NUF2 | 0.79 | KIFC1 | 0.81 |
| CENPI | 0.60 | TRIP13 | 0.67 | RAD54L | 0.73 | FAM72D | 0.76 | TOP2A | 0.79 | DLGAP5 | 0.81 |
| RDM1 | 0.60 | E2F8 | 0.68 | E2F2 | 0.73 | UHRF1 | 0.76 | CENPF | 0.79 | ASF1B | 0.81 |
| ZNF367 | 0.61 | RAD51 | 0.68 | FAM111B | 0.73 | MYBL2 | 0.76 | ESPL1 | 0.79 | CDCA8 | 0.81 |
| RAD51AP1 | 0.61 | CCNB2 | 0.68 | ARHGAP11A | 0.74 | MLF1IP | 0.76 | CDCA5 | 0.79 | SPC25 | 0.82 |
| CCNE2 | 0.61 | WDR62 | 0.68 | SHCBP1 | 0.74 | CENPE | 0.76 | NEIL3 | 0.79 | GTSE1 | 0.82 |
| ZNF695 | 0.61 | CCNB1 | 0.68 | DIAPH3 | 0.74 | BUB1B | 0.76 | RRM2 | 0.79 | NCAPG | 0.82 |
| KIF18A | 0.62 | CDC45 | 0.69 | OIP5 | 0.74 | BUB1 | 0.77 | HMMR | 0.80 | SKA3 | 0.82 |
| SGOL2 | 0.62 | FAM54A | 0.69 | ZWINT | 0.74 | BIRC5 | 0.77 | FOXM1 | 0.80 | AURKA | 0.83 |
| C11orf82 | 0.62 | PKMYT1 | 0.70 | CDCA2 | 0.74 | AURKB | 0.77 | ERCC6L | 0.80 | CDKN3 | 0.83 |
| GINS1 | 0.63 | PTTG1 | 0.70 | KIF23 | 0.74 | EXO1 | 0.77 | NEK2 | 0.80 | KIF4A | 0.83 |
| POC1A | 0.63 | UBE2T | 0.71 | KIF11 | 0.74 | KIAA0101 | 0.77 | KIF18B | 0.80 | IQGAP3 | 0.83 |
| ORC1L | 0.63 | MKI67 | 0.71 | RACGAP1 | 0.75 | PRC1 | 0.78 | CCNA2 | 0.80 | NUSAP1 | 0.83 |
| CENPM | 0.63 | ORC6L | 0.71 | KIF15 | 0.75 | ANLN | 0.78 | SPAG5 | 0.81 | PLK1 | 0.83 |
| E2F1 | 0.64 | CDCA3 | 0.71 | SGOL1 | 0.75 | DEPDC1B | 0.78 | SKA1 | 0.81 | CDK1 | 0.84 |
| FAM72A | 0.64 | DTL | 0.71 | ASPM | 0.75 | EPR1 | 0.78 | CEP55 | 0.81 | CDC25C | 0.84 |
| CIT | 0.65 | MND1 | 0.71 | CKAP2L | 0.75 | KIF2C | 0.78 | CDC20 | 0.81 | CENPA | 0.84 |
| LMNB1 | 0.65 | MCM10 | 0.71 | KIF14 | 0.75 | FAM64A | 0.78 | UBE2C | 0.81 | KIF20A | 0.84 |
| BLM | 0.66 | EZH2 | 0.72 | TACC3 | 0.75 | PBK | 0.78 | NCAPH | 0.81 | TPX2 | 0.85 |
| SPC24 | 0.66 | TK1 | 0.72 | POLQ | 0.75 | TROAP | 0.78 | MELK | 0.81 | HJURP | 1.00 |

The P value of above all lower than 0.05; ρ, Spearman rank correlation coefficient.
