# Supplementary figures and images for: HJURP promotes proliferation in prostate cancer cells through increasing CDKN1A degradation via the GSK3β/JNK signaling pathway
Source: Cell Death Dis. 2021 Jun 7;12(6):583. doi: 10.1038/s41419-021-03870-x (PMC8184824; doi:10.1038/s41419-021-03870-x)

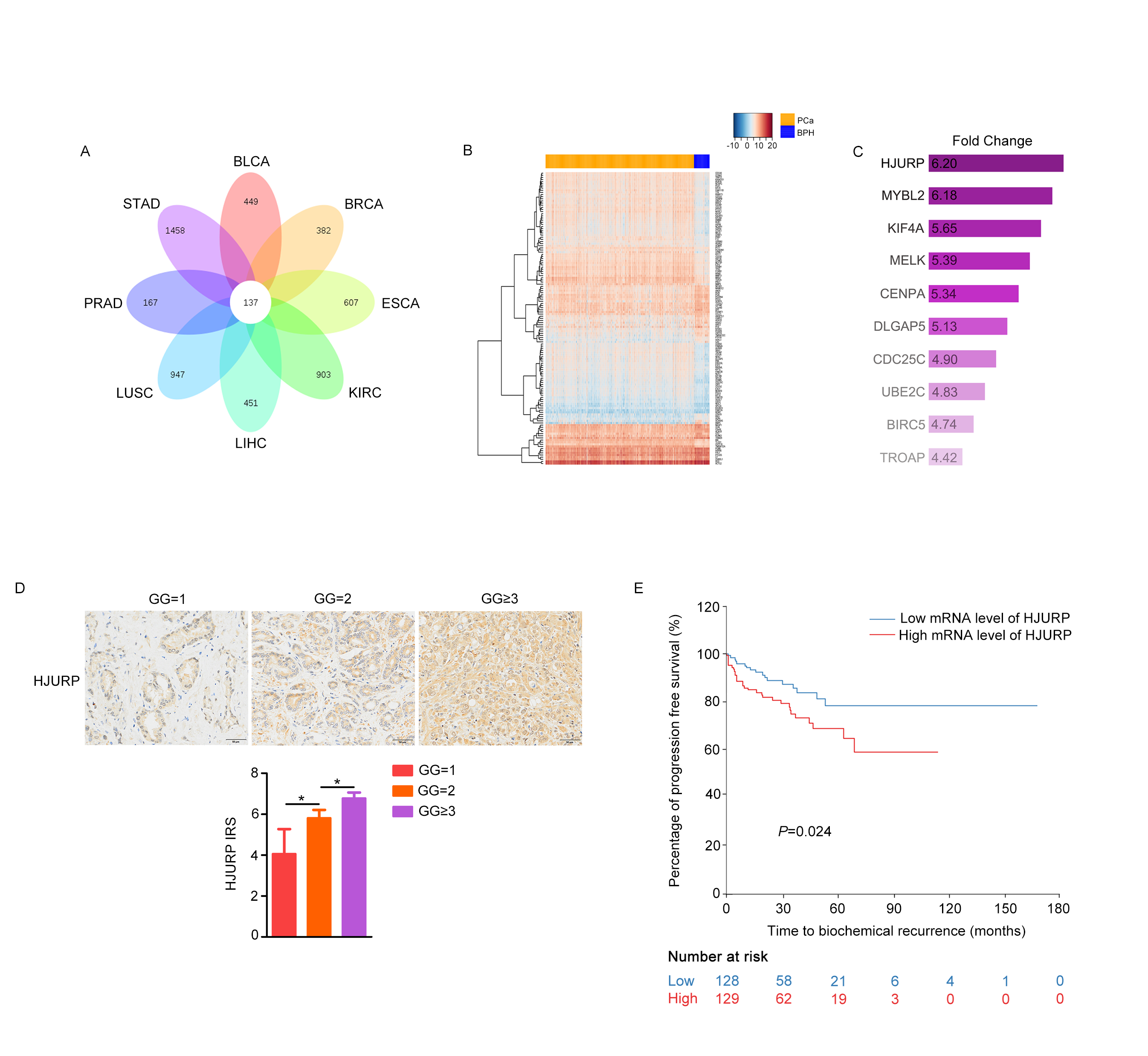

Supplement: Supplementary file 6 — Supplementary Fig. S1 [file 41419_2021_3870_MOESM6_ESM.tif]

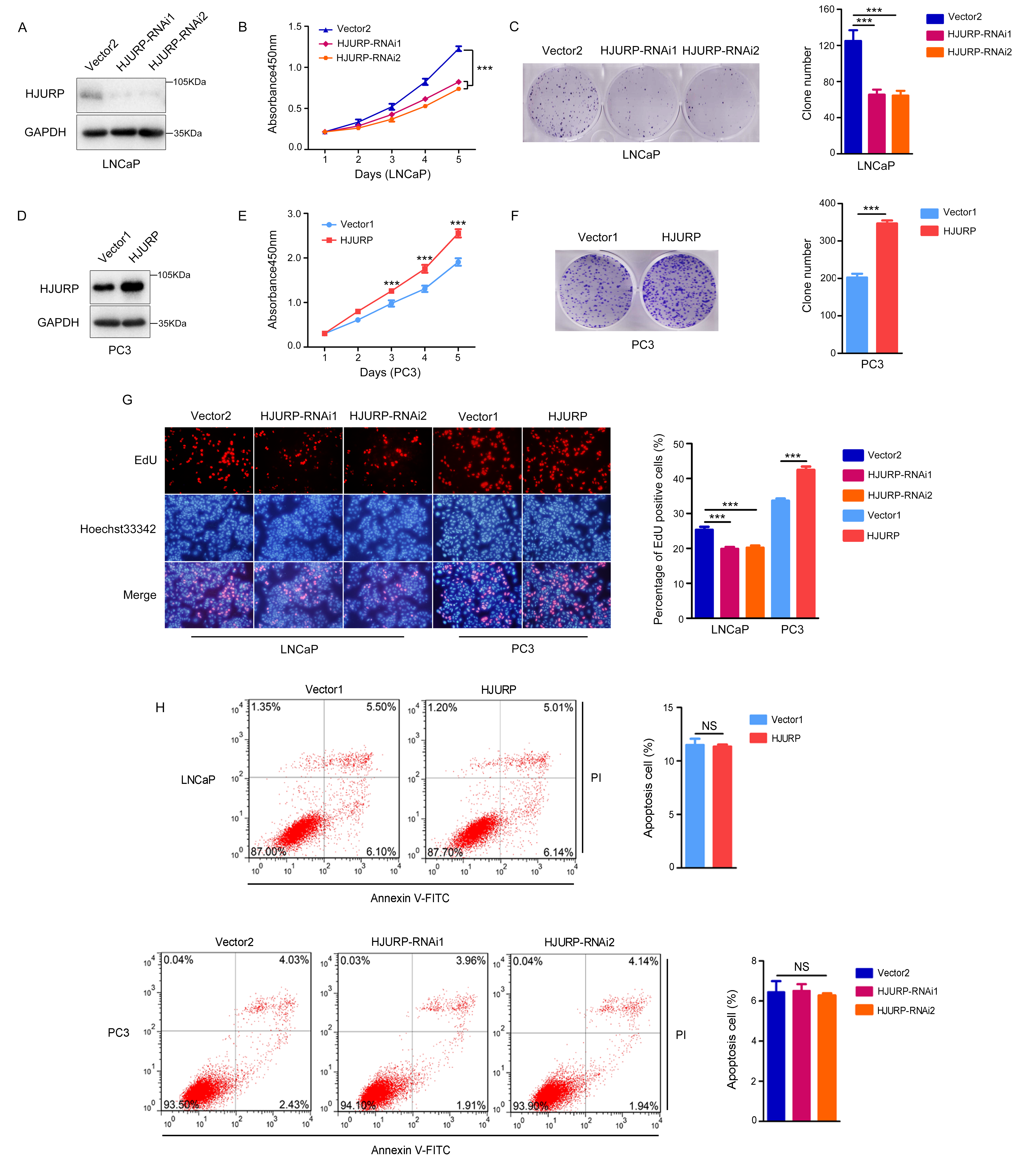

Supplement: Supplementary file 7 — Supplementary Fig. S2 [file 41419_2021_3870_MOESM7_ESM.tif]

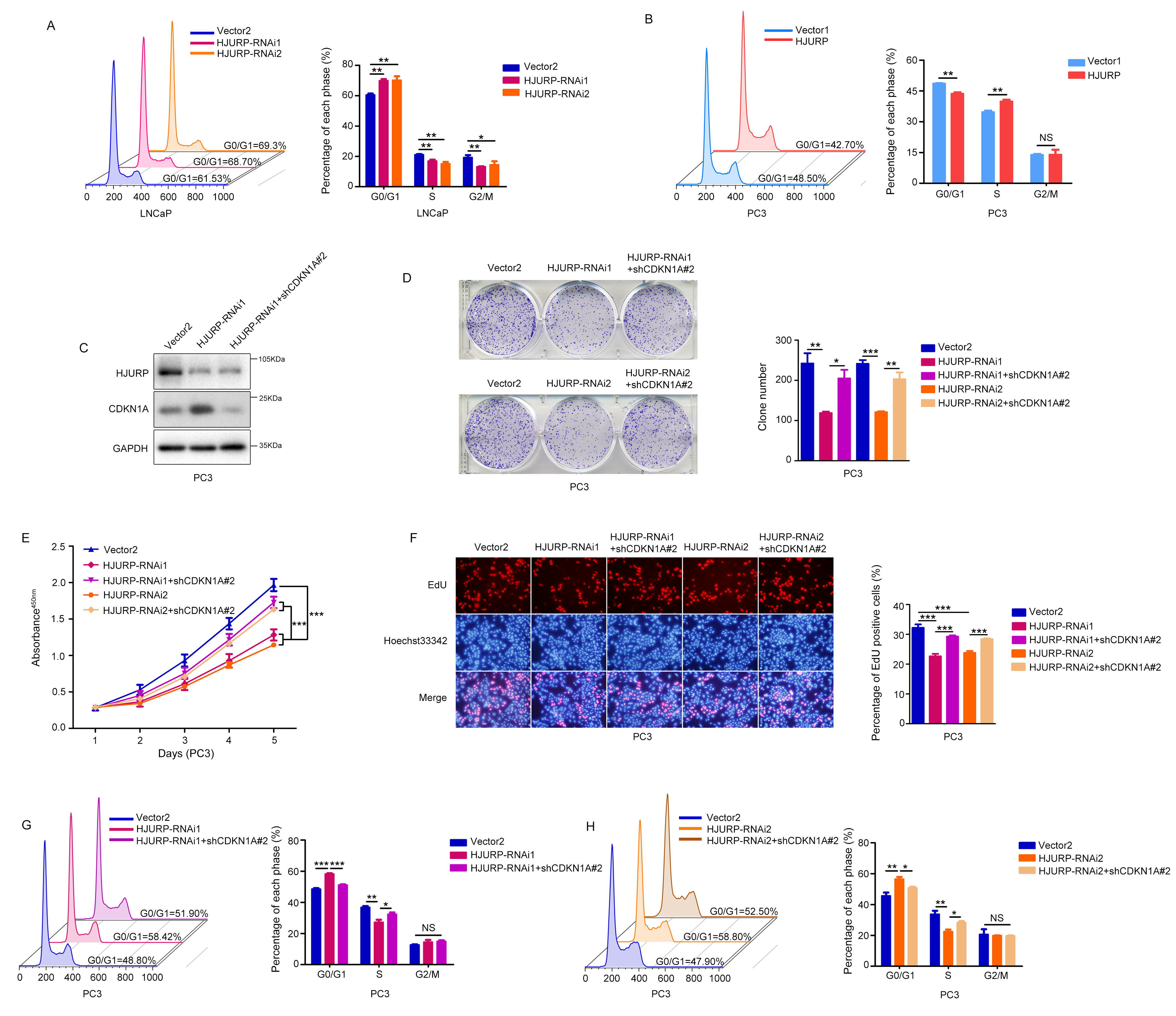

Supplement: Supplementary file 8 — Supplementary Fig. S3 [file 41419_2021_3870_MOESM8_ESM.tif]

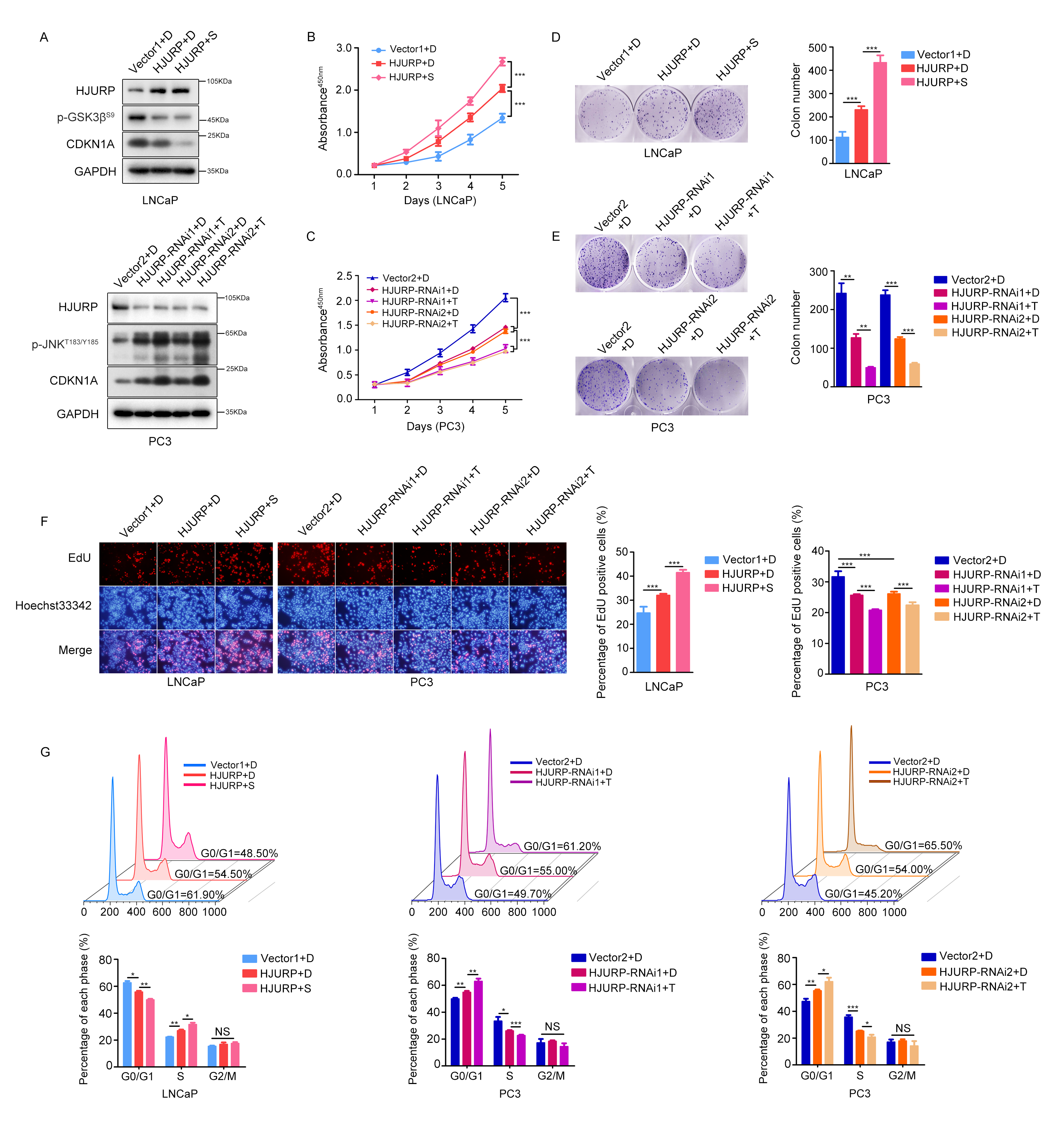

Supplement: Supplementary file 9 — Supplementary Fig. S4 [file 41419_2021_3870_MOESM9_ESM.tif]
