# Supplementary material for: HJURP promotes proliferation in prostate cancer cells through increasing CDKN1A degradation via the GSK3β/JNK signaling pathway
Source: Cell Death Dis. 2021 Jun 7;12(6):583. doi: 10.1038/s41419-021-03870-x (PMC8184824; doi:10.1038/s41419-021-03870-x)
Supplement: Supplementary file 10 — Supplemental Figure legends [file 41419_2021_3870_MOESM10_ESM.docx]

Fig. S1. A total of 137 cross-oncogenes were identified, of which *HJURP* was most significantly elevated in PCa, and high *HJURP* mRNA levels were associated with a short time to biochemical recurrence. A, the Venn diagram shows that there are 137 cross-oncogenes in all tumors. B, the heat map indicates differential distribution in 137 cross-oncogenes between PCa patients and controls. C, the top 10 genes differentially expressed in PCa, of which *HJURP* was most significantly elevated at 6.2-fold; D, Representative IHC staining of *HJURP* in different Gleason-grade PCa tissues from tissue microarray (scale bar: 50 µm; magnification: 200×); E, Kaplan-Meier plots demonstrating time to biochemical recurrence after radical prostatectomy stratified by *HJURP* mRNA levels. GG, Gleason grade.

Fig. S2. *HJURP* promotes proliferation but did not affect the apoptosis of PCa cells. A and D, western blotting of *HJURP*-knockdown (A) and overexpression (D) efficiency; B and E, CCK8 assay to measure PCa cells viability after *HJURP*- knockdown (B) and overexpression (E); C and F, colony formation assay to measure the cells viability of PCa cells after *HJURP* knockdown (C) and overexpression (F); G, EdU assay to measure the effects of *HJURP* on the proliferative capacity of PCa cells; H, Flow cytometry of PCa cells apoptosis.

Fig. S3. *HJURP* inhibits *CDKN1A* to promote G1/S phase transition. A and B, flow cytometry showed that HJURP promoted G1/S phase transition in PCa cells; C, western blotting results of *CDKN1A* knockdown efficiency; D-H, *CDKN1A* knockdown significantly reversed changes in clonogenic potential (D), cell viability (E), proliferative capacity (F) and cell cycle (G and H) in PC3 cells caused by *HJURP* inhibition.

Fig. S4. *HJURP* promotes the proliferation of PCa cells through the *GSK3β/JNK* pathway. A, SP600125 could assist in *HJURP*-induced *CDKN1A* inhibition, but did not affect *p-GSK3β^S9^* levels; TWS119 could enhance *HJURP*-induced upregulation in *p-JNK^T183/Y185^* and *CDKN1A*; B-G, TWS119 and SP600125 could promote *HJURP*-induced changes in cell viability (B, C), clonogenic potential (D, E), proliferative capacity (F), and cell cycle (G) in PCa cells.
