# Supplementary material for: HJURP promotes proliferation in prostate cancer cells through increasing CDKN1A degradation via the GSK3β/JNK signaling pathway
Source: Cell Death Dis. 2021 Jun 7;12(6):583. doi: 10.1038/s41419-021-03870-x (PMC8184824; doi:10.1038/s41419-021-03870-x)
Supplement: Supplementary file 11 — Supplementary Methods [file 41419_2021_3870_MOESM11_ESM.docx]

**Supplementary Methods**

**Inclusion criteria and exclusion criteria**

**The cohort from The Third Affiliated Hospital of Sun Yat-sen University**

A total of 843 patients with prostate cancer were treated in our hospital from June 2015 to December 2020, and 131 patients were selected according to the criteria for analysis.

The following inclusion criteria were used: (1) patients with radical prostatectomy; (2) surgical pathology confirmed prostate cancer; (3) complete clinical information, including age, preoperative PSA, Gleason score, pathological T and N stage, surgical margins, prostate volume.

The following exclusion criteria were used: (1) patients who were diagnosed by biopsy but did not undergo radical prostatectomy (n = 435); (2) patients whose prostate cancer lesions were found by biopsy, but not found by surgical pathology after radical prostatectomy (n = 59); (3) patients with incomplete clinical information mentioned above (n = 218).

This cohort was not followed up.

**The cohort from tissue array**

The tissue microarray contains samples from 159 patients with prostate cancer who underwent radical prostatectomy and were diagnosed by surgical pathology. All samples contain complete follow-up time and clinical data, including age, Gleason score, pathological T and N stage and surgical margins. But 9 sample tissues falling off during immunohistochemical analysis, 150 samples were analyzed finally.

The 150 patients were followed up from 0.85 to 8.92 years, and a total of 20 patients have died in this cohort by last follow up.

**The cohort from FireBrowse database**

Clinicopathological information from 499 prostate cancer patients was obtained from the FireBrowse database, and 257 patients were screened for analysis.

The following inclusion criteria were used: (1) patients with radical prostatectomy; (2) surgical pathology confirmed prostate cancer; (3) detailed follow-up time; (4) complete clinical information, including age, preoperative PSA, Gleason score, pathological T and N stage.

The following exclusion criteria were used: (1) patients without radical prostatectomy (n = 206); (2) patients with incomplete clinical data mentioned above (n = 36). For details of the extracted clinical information items, please see Supplementary Table S1.

The 257 patients were followed up from 0.08 to 13.88 years, and a total of 48 patients developed biochemical recurrence in this cohort by last follow up.

**Related computerized programs for the difference analysis using R in this study.**

**Separate tumor tissue from normal tissue according to the TCGA code.**

dat = read.table ("file.name", header = TRUE, sep="\t")

dim(dat)

library(stringr)

dat.tumor1 = dat2[,str_detect(colnames(dat2),'.01A')]

dat.tumor2 = dat2[,str_detect(colnames(dat2),'.01B')]

dat.tumor = cbind(dat.tumor1,dat.tumor2)

dim(dat.tumor)

dat.normal1 = dat2[,str_detect(colnames(dat2),'.11A')]

dat.normal2 = dat2[,str_detect(colnames(dat2),'.11B')]

dat.normal = cbind(dat.normal1,dat.normal2)

dim(dat.normal)

dat.final = cbind(dat.tumor,dat.normal)

write.table(dat.final,"datExp1.txt",col.names=TRUE,row.names=TRUE,quote=F,sep="\t")

**The RNA-seq data were normalized.**

dat.exp = read.table("datExp1.txt",header=TRUE,sep="\t")

dat.exp[dat.exp == 0] = 1

dat.exp = log(dat.exp,2)

write.table(dat.exp,"dat-log1.txt",col.names=TRUE,row.names= TRUE,quote=F,sep="\t")

pheno = NULL

pheno$sample = colnames(dat.exp)

pheno$group = c(rep('tumor',ncol(dat.tumor)),rep('normal',ncol(dat.normal)))

pheno = as.data.frame(pheno)

head(pheno)

**The RNA-seq data were analyzed using the “limma” package to find differential genes between tumor and normal.**

library(limma)

Group = factor(pheno$group,levels=c('tumor','normal'))

design = model.matrix(~0+Group)

colnames(design) <- c('tumor','normal')

design

fit <- lmFit(dat.exp, design)

contrast.matrix <- makeContrasts(tumor-normal, levels=design)

fit2 <- contrasts.fit (fit, contrast.matrix)

fit2 <- eBayes(fit2)

all.deg = NULL

diff=topTable(fit2,adjust.method="fdr",coef=1,p.value=0.05,lfc=log(2,2),number=50000,sort.by = 'logFC')

write.table (diff,'DEG.txt',col.names=TRUE,row.names=TRUE,quote=F,sep="\t")

diff = topTable(fit2,adjust.method="fdr",coef=1,,number=50000,sort.by = 'logFC')

diff 0 = diff

P.value = diff0$adj.P.Val

FC = diff0$logFC

df <- data.frame(P.value,FC)

df$threshold = as.factor(abs(df$FC) > log(2,2) & df$P.value < 0.05)

levels(df$threshold) = c('grey','red')

df$logp = -log10(df$P.value)

plot(x = df[,2],y = df[,4], pch=16, col=df$threshold,

xlab="Fold change", ylab="-Log10(Pvalue)",

cex=0.5, main="Volcano Plot")

abline(v=c(-log(2,2),log(2,2)), h=-log10(0.05),col="green")

**Intersecting the differential genes of eight tumors using Venn diagram (https://www.omicshare.com/) to find Cross-genes.**

**Related computerized programs for the analysis of clinical data using Perl or R in this study.**

**According to the TCGA code, the clinical data of PCa patients were matched with their RNA-seq data using Perl.**

use strict;

chomp(my $file1=shift);

chomp(my $file2=shift);

open FILE1,'<',$file1;

open FILE2,'<',$file2;

my(@common,@deg,@temp,$p,$b,$b2);

while(<FILE1>){

chomp;

push(@common,$_);

}

while(<FILE2>){

chomp;

push(@deg,$_);

}

foreach $b (@deg){

chomp;

@temp = split /\s+/,$b;

foreach $p (@common){

if($p eq $temp[0]){

print"$b\n";

}

}

}

**Based on the median of HJURP, patients were divided into high-level and low-level groups for subsequent analysis.**

**Survival analysis using R**

dat=read.table('file.name',sep='\t',header=TRUE)

library("survival")

library("survminer")

fit<-survfit(Surv(times,status)~HJURP,data=dat)

print(fit)

plot(fit)

survdiff(Surv(times,status)~HJURP, data=dat,rho = 1)

survdiff(Surv(times,status)~HJURP, data=dat,rho = 0)

**SPSS was used for other statistical analyses in this study**
